# Impacts of English language proficiency on healthcare access, use, and outcomes among immigrants: a qualitative study

**DOI:** 10.1186/s12913-021-06750-4

**Published:** 2021-07-26

**Authors:** Mamata Pandey, R. Geoffrey Maina, Jonathan Amoyaw, Yiyan Li, Rejina Kamrul, C. Rocha Michaels, Razawa Maroof

**Affiliations:** 1 Research Department, Wascana Rehabilitation Centre, Saskatchewan Health Authority, 2180-23rd Ave, Regina, SK S4S 0A5 Canada; 2 College of Nursing, University of Saskatchewan, Prince Albert, SK Canada; 3 Department of Sociology and Social Anthropology, Dalhousie University, Halifax, NS Canada; 4 Department of Academic Family Medicine, University of Saskatchewan, Regina, SK Canada

**Keywords:** Language barriers, Immigrants, Healthcare access, Healthcare utilisation, Health outcomes

## Abstract

**Background:**

Immigrants from culturally, ethnically, and linguistically diverse countries face many challenges during the resettlement phase, which influence their access to healthcare services and health outcomes. The “Healthy Immigrant Effect” or the health advantage that immigrants arrive with is observed to deteriorate with increased length of stay in the host country.

**Methods:**

An exploratory qualitative design, following a community-based research approach, was employed. The research team consisted of health researchers, clinicians, and community members. The objective was to explore the barriers to healthcare access among immigrants with limited English language proficiency. Three focus groups were carried out with 29 women and nine men attending English language classes at a settlement agency in a mid-sized city. Additionally, 17 individual interviews were carried out with healthcare providers and administrative staff caring for immigrants and refugees.

**Results:**

A thematic analysis was carried out with transcribed focus groups and healthcare provider interview data. Both the healthcare providers and immigrants indicated that limited language proficiency often delayed access to available healthcare services and interfered with the development of a therapeutic relationship between the client and the healthcare provider. Language barriers also impeded effective communication between healthcare providers and clients, leading to suboptimal care and dissatisfaction with the care received. Language barriers interfered with treatment adherence and the use of preventative and screening services, further delaying access to timely care, causing poor chronic disease management, and ultimately resulting in poor health outcomes. Involving untrained interpreters, family members, or others from the ethnic community was problematic due to misinterpretation and confidentiality issues.

**Conclusions:**

The study emphasises the need to provide language assistance during medical consultations to address language barriers among immigrants. The development of guidelines for recruitment, training, and effective engagement of language interpreters during medical consultation is recommended to ensure high quality, equitable and client-centered care.

## Background

Major immigrant-destination countries like the United States, Germany, Canada, and Australia admit a large share of immigrants from culturally and linguistically diverse countries [1]. According to the 2016 Canadian Census, foreign-born individuals make up more than one-fifth (21.9%) of the Canadian population, which is close to the highest level (22.3%), recorded in the 1921 Census [2]. Most immigrants to Canada come from countries like the Philippines, India, China, Nigeria, and Pakistan, where most citizens’ first language is neither English nor French [3–5]. Individuals without local language proficiency are more likely to have lower income, and face considerable challenges with economic and social integration [6–8]. These settlement challenges increase the risk of poor health outcomes among newcomers with limited language proficiency [9]. Newcomers also face inequities in healthcare settings [10]. Due to immigration requirements, most newcomers are healthier than the general population, an effect referred to as the “healthy immigrant effect.” This effect is observed to decline over time [11–13]. Limited language proficiency is associated with decline in self-reported health status of new immigrants during the first 4 years of stay in Canada [9].

The ability to speak the host country’s official language proficiently appears to be an essential determinant of health [13–16]. The ability to speak, read, and write in the local language is necessary to communicate with healthcare providers and interact in other social settings [17–19]. Language is consistently identified as a barrier for immigrants and refugees seeking, accessing, and using mental health services [11, 12, 15, 20]. Lee and colleagues [21] argued that Chinese immigrant women are more likely to choose service providers who speak the same language. Marshall, Wong, Haggerty, and Levesque [4] observed that Chinese- and Punjabi-speaking individuals with limited English language proficiency might delay accessing healthcare to find providers who speak their language. In the absence of culture-specific words and due to stigma, individuals from some ethnics groups may have difficulty describing mental health conditions or describe them as somatic symptoms [12, 22–24]. Lack of language support or culturally appropriate services can interfere with timely mental health diagnosis and/or utilization of mental health services [12, 23, 24].

Language-incongruent encounters within the healthcare system increase the risk of inadequate communications, misdiagnosis, medication errors and complications, and even death [15, 19, 25]. Studies indicate that language barriers adversely affect health outcomes, healthcare access, utilization and cost of healthcare services, health-providers’ effectiveness, and patient satisfaction and safety [15, 25–33].

Aery and colleagues [34] argue that the rights that allow individuals access to language interpreters in the justice system are also applicable in the healthcare context. Without language assistance, individuals with language barriers cannot engage in their treatment, determine risks and benefits of suggested treatment, and/or provide informed consent [34, 35]. Human rights legislations in Canada have provided a framework and highlight the necessity to provide language interpreters when needed, but these have not been implemented universally [35]. Some provinces in Canada have launched language interpretation services. These services include: the Language Services Toronto in Ontario, language services for French-Canadians offered by Winnipeg Health Region in Manitoba and CanTalk telephonic interpreter services approved by the Saskatchewan Health Authority in Saskatchewan [35–38]. Professional interpreter services are not covered under most provincial health policies and therefore might not be available in all jurisdictions [3]. In the absence of universal interpretation services across the country, healthcare providers rely on professional interpreters, interpreters from community-based organizations and/or ad hoc (untrained) interpreters such as family members, friends, and volunteers who lack understanding of medical terminology and disease [3, 36–38]. Although the services of professional language interpreters are employed in many Canadian healthcare settings, reliance on ad hoc interpreters, is preponderant [35]. This is partly due to a lack of trained interpreters in the language required and new immigrants’ lack of knowledge about available language supports [10]. Providers are also not comfortable with interpreters as it is time consuming, and providers might have different expectations about the roles of interpreters [3]. The impacts of local language proficiency on immigrants’ health and well-being are relevant and have been studied in other major immigrant-destination countries such as Australia, the United Kingdom, the United States of America [15, 17, 25, 32].

This topic is particularly relevant in the Canadian context as 72.5% of immigrants are reported to have a mother tongue other than English or French according to the 2016 Census [39]. Given the unique history, culture, ethnic composition, and organization of healthcare services in Canada, scholars have highlighted the need for Canadian-based studies exploring how language barriers contribute to inefficiencies within the Canadian healthcare system and what strategies can be developed to address the gaps [10, 15]. This study explores the impact of language barriers at each point of contact with the healthcare delivery system, from the perspective of immigrants and healthcare providers in a Canadian province that is witnessing a rapid influx of immigrants [2]. Taking a comprehensive approach, the study examined the overall impacts of language barriers on healthcare access, satisfaction with care received and health outcomes.

## Methods

The study was set in a mid-size prairie city. An exploratory qualitative research approach guided by the principles of community-based research methods was adopted. Clinicians on the research team experienced many challenges while caring for both immigrants and refugees with language barriers. These clinicians approached community members for their perspective. The study idea was conceived after collective brainstorming with multi-sectoral stakeholders, including: representatives from a non-government settlement agency providing various settlement services to immigrants, family physicians caring for both immigrants and refugees in the city, and health researchers. Each stakeholder represented a specific ethnic-minority group and arrived in Canada as a landed immigrant. Through personal experiences and professional interactions with other immigrants, the stakeholders knew about barriers experienced during healthcare access.

Thereafter, stakeholders developed a research partnership. They collectively decided to document these challenges and leverage the research results to advocate for improved healthcare services. The study aim was to explore the perspectives of immigrants and of healthcare providers. Other groups, such as temporary migrant workers and refugees, have other unique challenges not within the scope of the study. Community partners assisted the research team to finalize the research question and determine methods of participant recruitment. The study was carried out in two parts and approved by the provincial health authority’s research ethics board (REB 14–122 and REB 15–69).

### Part 1

#### Participants

A purposeful sampling method was used. Community partners assisted with participant recruitment by engaging those seeking services through a settlement agency. All participants recruited were immigrants. The consent form and roles of research participants were shared with all 43 individuals attending English language classes at the settlement agency. Language assistance was provided by interpreters and the English language teachers facilitating the classes. Thirty-seven individuals (28 female and nine male) from 15 different countries signed consent forms. Three participants were travelling, two just began English language classes and one participant was not interested and were excluded. All participants lived in Canada for less than 6 years and are hereafter referred to as “clients.” Please refer to demographic information of clients in Table 1.

**Table 1:** Sociodemographic information and current health information recorded from participants, by gender

	Female	Male
**n (%)**	28 (75.7%)	9 (24.3%)
**What is your Age? (M,SD)**	37.6 (7.9)	41.1 (7.1)
**What is your Marital Status? n (%)**		
*Married or common law*	25 (89.3%)	8 (88.9%)
*Divorced*	1 (3.6%)	1 (11.1%)
*Missing*	2 (7.1%)	N/A
**What level of Education you completed? n (%)**		
*Elementary Schooling (grade 10)*	1 (3.6%)	1 (11.1%)
*High school*	5 (17.9%)	5 (55.6%)
*Trades and or vocation*	1 (3.6%)	1 (11.1%)
*Undergraduate*	18 (64.3%)	2 (22.2%)
*Missing*	3 (10.7%)	N/A
**How many child you have? n (%)**		
*0*	5 (18%)	N/A
*1–3*	20 (71%)	8 (88.9%)
*> 3*	2 (7.1%)	1 (11.1%)
*missing*	1 (3.6%)	N/A
**How many other individuals live with you in the same house? n (%)**		
*1–3*	17 (60.7%)	3 (33.3%)
*4–6*	10 (35.7%)	3 (33.3%)
*> 6*	N/A	1 (11.1%)
*Alone*	N/A	1 (11.1%)
*Missing*	1 (3.6%)	1 (11.1%)
**What is the total family income in a year? n (%)**		
*0-$30,000*	9 (32.1%)	1 (11.1%)
*$30,000–$50,000*	6 (21.4%)	2 (22.2%)
*$50,000–$100,000*	4 (14.3%)	3 (33.3%)
*Missing*	9 (32.1%)	3 (33.3%)
**How long have you stayed in Canada?: Mean (*SD*) years**	2.8 (1.9)	3.4(.15)
**Continent of origin n**		
Asia (*Afganistan, China, India, Pakistian, Phillipines, Russia, and South Korea***)**	22	
Europe (*Hungary, Poland, Turkey and Ukraine*)	10	
Africa (Egypt, Tunisia, Eretria)	4	
South America	1	
**Current Health Status**		
***How is your health at present?***		
Good	18 (64.3%)	6 (66.7%)
Alright	10 (35.7%)	3 (33.3%)
Bad	N/A	N/A
***Do you get tired easily?***		
Yes	6 (21.4%)	2 (22.2%)
No	4 (14.3%)	3 (33.3%)
Sometimes	18 (64.3%)	4 (44.4%)
***Do you have problems with your sleep?***		
Yes	18 (64.3%)	4 (44.4%)
Sometimes	7 (25%)	3 (33.3%)
No	3 (10.7%)	2 (22.2%)

#### Data collection

The focus group discussion (FGD) questions were developed in consultation with the settlement agency staff and focused on: a) the clients’ perceptions of health and the services needed to stay healthy; b) differences between the healthcare systems in the client’s country of origin and Canada; c) access to healthcare services; d) challenges clients faced when accessing care in Canada; and, e) how clients made decisions about healthcare. Clients received the questions before the FGD to organize their thoughts. Medical students representing specific ethnic groups and speaking an additional language assisted with data collection and interpretation during the FGDs.

Three FGDs were held at the settlement agency and lasted 2 h with breaks for refreshments. Each FGD was attended by 10–15 clients and subgroups of 3–4 clients were coordinated by a facilitator speaking the same language. Clients with language barriers were supported by facilitators speaking their language, other clients with advanced English language proficiency, or language interpreters.

Responses from clients were written down by facilitators and reread to the clients for accuracy. Some clients had written down their thoughts in English using online translators prior to the actual FGD to help them verbalise their thoughts with ease. Clients read out their responses during the FGDs and handed in those written notes after the FGDs. Facilitators also wrote field notes of the salient points emerging from these sessions and their reflections, which informed subsequent FGDs. None of the clients received services from any of the family physicians on the research team during data collection. Complementary child minding, light refreshments and a $20 gift card to a grocery store were provided as incentives to participate.

### Part 2

#### Participants

In part 2, healthcare providers’ perspectives on caring for immigrants and refugees were explored to show a more comprehensive view of the situation. Seventeen healthcare providers and health administrative staff signed the consent form: four family physicians, two family physicians providing obstetrical care, a psychiatrist, a registered nurse, a lab technician, a pharmacist, a nutritionist, a psychiatric social worker, a counsellor, an exercise therapist, an ultrasound technician, an executive director, and a receptionist. They were recruited from a community clinic that predominantly served refugees, immigrants, and other socio-economically disadvantaged populations in the city, other medical clinics in the city, and a hospital.

#### Data collection

Healthcare providers serving immigrants and refugees participated in an hour-long, in-depth individual interview focusing on a) health services required to better address the healthcare needs of immigrants and refugees; b) the availability of culturally-responsive healthcare services; and c) the barriers to providing such care. Family physicians on the research team with extensive experience caring for immigrants and refugees assisted with the development of the interview guide and data collection. Interviews were carried out in English and were audio recorded. No compensation was provided.

### Analysis

The FGDs and healthcare provider interviews were transcribed verbatim. Data was analyzed qualitatively using NVivo version 9 following the procedure proposed by Miles, Huberman, and Saldana [40, 41]. During preliminary data analysis, two rich transcripts were open coded by a team of researchers. Although the project was carried out to explore barriers to healthcare access for immigrants, language barriers emerged as a distinct theme impacting various aspects of care during data analysis. The results were shared with the settlement agency representatives. A collective decision was made to highlight the impacts of limited English language proficiency on healthcare access, utilization, and outcomes for immigrants in this manuscript. This framework guided the rest of the data analysis. The research team collectively reviewed the completed data analysis report and no new themes emerged at this discussion. The research team collectively agreed that further clarifications were not required from participants. Therefore, follow-up focus groups or interviews were not carried out and no new participants were recruited..

**Fig. 1:**
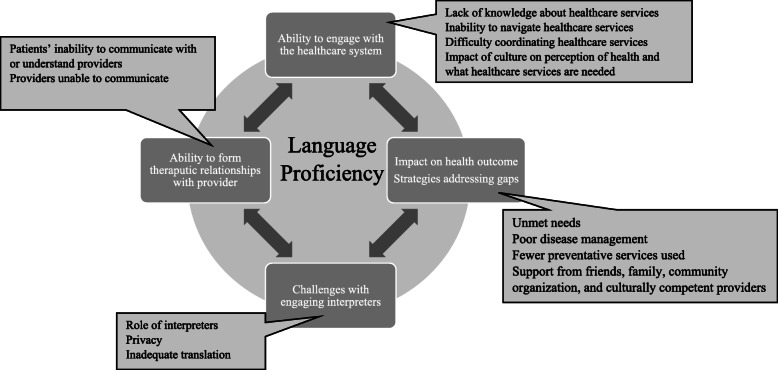
Language Proficiency Leads to Poor Healthcare Access, Suboptimal Care, and Dissatisfaction with Care

Data was broken into 120 base-level codes. The base-level codes were reviewed a second time, and codes with similar concepts were consolidated into 45 intermediate codes. The intermediate codes were categorized under 11 sub-themes. Title was assigned to each sub-theme to highlight the diverse and pertinent concepts represented by each sub-theme. The sub-themes were then organized under four central themes. Diagrammatic representation shows the relationship between the 11 sub-themes and the four themes and is illustrated in Fig. 1. Field notes maintained by facilitators were used to cross-reference the themes emerging during data analysis to ensure all pertinent themes were included. The diagram demonstrating the relationship with the subthemes was approved by all team members.

## Results

Impacts of limited English language proficiency have been summarized under four main themes as follows.

### Theme 1: ability to access health information and services

Language proficiency significantly impacted a client’s ability to identify services needed, to secure appointments, and to effectively engage with healthcare providers while seeking care and managing post-appointment care and follow-up. Information about healthcare services is usually provided in English or French. Thus, a client with language barriers lacked adequate information about available services and was unable to access services promptly. Clients with language barriers are less likely to actively seek health and/or mental health services when needed, as is evident from a client’s comment: “*No do not know about mental health services because of the language problem. Can I go to the hospital to access it?” [client].* Another client inquired: “*Do I need appointments for blood tests?*”

The range of healthcare services offered in different countries differs significantly. Lack of knowledge about existing healthcare services in the city created a barrier, which was greatly influenced by clients’ local language proficiency. A healthcare provider in the study commented that,

*“We need to make the community or the clients’ population know that this is available for you and this is the process how you get access to this service, the language barrier is a huge barrier for this population and to access like any health care service.”*

The way in which healthcare is organized and coordinated varies from country to country, and for newcomers, understanding the services provided within the host country largely depends on their ability to decipher information about them. Those with language limitations might not know how to access various healthcare services. This can lead to misunderstanding between the client and the provider, causing frustrations and unfulfilled expectations for both, as one healthcare provider noted:

*“I offer free prescription delivery, but clients didn't come to the door, they didn't understand that the delivery person is delivering it and all they're doing is going to the door, ringing the doorbell expecting them to be let in. On numerous occasions, we were unsuccessful because they [clients] wouldn't open the door, there was no one there or-they did not understand, so, unless someone on the other end speaks English and tells us they're going to be there, we won't deliver now.”*

Experience with healthcare delivery in clients’ countries of origin and cultural beliefs about health and what healthcare services should be accessed can interfere with their healthcare access. Language barriers may impede a client’s ability to understand the differences between healthcare organization in Canada and in their country of origin, leading to the underutilization of healthcare services, as one healthcare provider explained:

*“If you don't know their language, it becomes difficult to provide care to them. Also, cultural beliefs can interfere with access to care. For example, they [immigrants and refugees with language barrier] do not know how to access an optometrist or dentist. So, I have to give them a lot of information as they have no idea.”*

Due to language barriers, clients experienced difficulty following conversations with receptionists, providing proper documentation required for coordinating care, and booking and attending appointments. Clients with language barriers were less likely to seek clarifications when they did not understand instructions or to advocate for their needs. As one client noted, *“I don’t speak good English. Therefore, sometimes it is difficult to understand what the receptionist is saying.”*

Similarly, a health administrative staff mentioned *“I am still waiting for the healthcare number from three clients. They [clients with language barrier] do not understand it is necessary for billing purposes*”.

The degree to which clients with limited language proficiency are able to access the healthcare services they need largely depends on their ability to understand information that is written in English and to understand how the healthcare system is organized.

### Theme 2: ability to develop a therapeutic alliance with healthcare providers

English language proficiency significantly affected the therapeutic relationship between patients and healthcare providers. Clients with language barriers were unable to explain their health conditions adequately, as one client noted:

*“Without proficiency in English, it is difficult talking to the health care provider. It's a problem to describe what you're feeling. It will be easier as a newcomer if they have a family doctor who speaks the same language. Like for children with pain, it is difficult for them to say what they [children] want or to make them [children] understand*.”

Clients reported experiencing difficulty asking questions about their health and understanding treatment instructions. One client mentioned that,

*“Sometimes, the doctors describe the illness in a way that I don’t understand what the doctors say. Sometimes this makes it very hard to go to the doctors because of the language problems.”*

Healthcare providers were often concerned about not getting adequate information about health concerns from patients with language barriers. They experienced difficulties during physical examinations or when providing treatment instructions, which can have adverse outcomes, as one healthcare provider explained:

*“Say I am treating an ear infection. I have told the clients many times that the medication is to be administered by mouth, but they thought it was to be installed in the ears. So, I have a couple of disastrous cases where I have prescribed medication where they don’t realize it is given by mouth. I think also, when they don’t understand, they feel uncomfortable to ask for clarification. They get very embarrassed and they get very frustrated.”*

Similarly, clients with English language barriers also mentioned difficulty understanding medication regime as a client mentioned.

*“I had problems with the iron levels, the doctors prescribed iron pills. I asked the doctors how many to take, but he did not explain it properly. He first said that I should take one pill a day, then when I ask if that will be enough, he said I can take 2 to 3 pills. How can he advise me like that without explaining it properly?”*

### Theme 3: challenges with engaging language interpreters

Language interpreters are not available at all clinics and families often bring ad hoc interpreters to the appointments or use volunteers working within the healthcare system. Often, these ad hoc interpreters lack adequate skills and training to carry out medical translation, which creates additional challenges. Healthcare providers may not feel confident that instructions are being translated verbatim. They also noted that often they received a summarized or concise version of what the clients narrated and wondered whether valuable contextual information was lost during translation. This can be frustrating for the healthcare providers and interfere with the development of the therapeutic alliance, as a healthcare provider pointed out:

“Some of the barriers I've experienced, those mainly had to do with communication and interpreters. I guess sometimes I wonder with the translation, what is being said to the patient. because they have quite a long discussion, and then when I ask the interpreter what was said … oh, they have no questions. *laughs* so I'm not sure what the conversation was, so that can be a little bit, um, frustrating.”

Further, some interpreters might provide a cultural and/or religious interpretation of strategies that might not align with Western medical care, as this healthcare provider explained:

*“There are times when the clients will bring in their interpreters that I don't feel that my teaching and my advice is being given to them appropriately or word for word. I find that the personal interpreters they bring in will contraindicate and conflict with what I am telling the client because they will say "no that's not how we do things" instead of telling the client what I as a practitioner would like them to do”.*

Sometimes, ad hoc interpreters are less helpful in assisting with client-provider communication and they may become an impediment to the therapeutic alliance, as a healthcare provider noted:

*“Sometimes working with an interpreter is difficult because you don't always know whether the translator translates exactly what you're trying to come across or explain.”*

Some clients were also concerned that their messages were not communicated properly to the healthcare providers during translation as a client mentioned:

“*I cannot speak English so I cannot go by myself to the doctor … … Before I had to wait for my husband he works, and say everything fast as he had to go back to work soon, I could not say everything I wanted, to the doctors, but now my son comes with me so it is better but I have to remind him always to say everything I said, to the doctor as he is still young and may forget*.”

A medical interpreter’s presence can create privacy and confidentiality issues, especially for clients with mental health issues. Interpreters assisting clients with mental illness require training to create culturally safe interactions, lest the interaction become more injurious to the clients than the illness itself. The excerpt below from a healthcare provider is an excellent example of culturally unsafe medical translation.

*“I had this case where the interpreter was not trained in mental health, and they found the conversation to be funny, so it was an elderly Asian lady who had delusions and hallucinations—well, we had a hard time with that. The interpreter was laughing.”*

Some clients were uncomfortable receiving language assistance from family or individuals of the same community. As is mentioned by a women client:

“*I need lady doctor or lady speaking my language. I need medicine to stop baby [contraception] where can I get it. I cannot talk about this with my doctors when others [family members who help with translation] are there with me and I am waiting for 3 months now.”*

Moreover, healthcare providers were sometimes concerned about the quality of the translation services provided to their clients. Healthcare providers observed that some interpreters struggled to explain instructions adequately during sample collection and diagnostics tests, leading to delays in the treatment process and linkage to treatment. One healthcare provider conveyed the issues with inadequate medical translation:

*“I requested that the client present with a stool sample in the container provided. A couple of times, some clients showed up with urine in there rather than stool. This is after numerous explanations with an interpreter present.”*

Another healthcare provider mentioned that:

*“Giving simple instructions such as the need for a full bladder before ultrasound, many don’t understand what bladder is. Last week I tried to conduct spirometry on a patient even with the presence of an interpreter and I was not successful. He just didn't understand. I guess he [interpreter] did not translate accurately.”*

Effective communication between healthcare providers and clients is vital for providing safe and quality healthcare.

### Theme 4: impacts of language barriers on health outcome and strategies addressing gaps

Clients with language barriers often manage care on their own and due to lack of effective communication they are often dissatisfied with care received. Clients felt as though it was not worth seeking care when there was no means of addressing their language limitations, as one client noted:

*“This country has so much resources and sometimes I feel the resources are not put to good use*. *What is the point of seeing a doctor if I do not feel satisfied? First, you must make appointments, manage everything at home to go for that appointment, and then still wait when you reach there, and then the doctors hardly spend time with you.”*

In many countries healthcare is accessed on a need to basis and individuals might not have understanding about preventative health. Emphasis is given on preventative medicine in Canada, but providing health education can be challenging due to language barriers as a healthcare provider pointed out:

*“If they don’t understand the preventative or the treatment plan but instead of perhaps doing some preventative stuff, they want to jump right to the surgery or jump right to the medication. Like PAP smears and mammograms, there is a lack of education in those countries where they come from. There are no concepts of preventative health care there. We tried to offer an information session with interpreters it really slowed down the meeting; everyone had to wait for the interpreter to interpret our directions and if we didn’t immediately have them interpret the participants were having a hard time following the conversation”*

Healthcare providers were apprehensive about the dangers that clients with language barriers might face away from healthcare setting, as was explained by this healthcare provider:

*“First of all, they [clients] might not understand what I'm telling them when I'm asking them to administer insulin themselves and increasing their doses based on their numbers. A lot of times they’re very confused on that fact and the translation, something is getting lost in the translation. Any misunderstanding can put them in a very dangerous situation if they give themselves too much insulin.”*

Language ability can interfere with chronic disease management, which requires continual monitoring through regular clinic appointments. Even with medical translation, some clients may not comprehend the steps in the treatment plan that they are required to follow to manage chronic conditions effectively. Without additional supports available after medical appointments, these patients struggle to set up follow-up appointments, refill prescriptions, and adhere to medical instructions. In the absence of supports, treatment adherence might be poor. A healthcare provider describes what happens when clients don’t receive post-appointment follow-up or support:

*“A lot of them [clients] have chronic conditions such as hypertension and don't come for a routine check-up. You'll see them and start them on medication and try to emphasize that this is long term treatment, and they will need to come back in a month for a check-up. You'll see that they've shown up a year later, and yet they were prescribed medications to last them for one month only and didn't renew them even though they had renewals. They will show up a year later with a headache or something, and their blood pressure is way out of control. I see that a lot.”*

Clients mentioned adopting few strategies to address language barriers. Women clients often preferred same gender interpreters for women health issues and they depended on family and friend circles for assistance as a client mentioned: “*I have a very good friend who took holiday from work to come with me, I had to talk to the doctor about women problem*.” Clients also consulted friends or family to find relevant healthcare services near them. A client mentioned: *“I will ask my sister for healthcare for my family she and her family help us when we need information. I can also find out using the internet.”* Clients might also seek information about healthcare services and ways to access it from community organizations providing settlement services as a client mentioned: “*I ask my English teacher when I need information about healthcare services they can help me.*”

Some clients pointed out that finding providers from their ethnic background would be helpful. Many clients take it upon themselves to seek care from these providers and may delay healthcare access, as this participant mentioned: *“I am waiting to find a doctor who speak my language and can understand my culture.”* Matching clients with providers from the same linguistic and ethnic background is useful but challenging. It may be more feasible in larger cities with larger and established ethnic groups. A client who received care from a provider from the same ethnic background mentioned a positive experience, as is evident from this comment:

*“My doctor is from my country and he was able to explain to me why I need the surgery (hysterectomy). I was scared and I did not want to do it, but my husband and my doctor helped me understand that it was needed and if I did not get it done I will get very sick, I did it and I am alright now.”*

Alternatively, healthcare providers who are culturally attuned to the challenges that clients with language barriers face are often empathetic and accommodative and ensure that clients receive the required care. One healthcare provider noted:

*“They experience barriers accessing health care due to language limitations. Some clients may have challenges with conceptualizing what constitutes good health. This is partly informed by the fact that most of them may have experienced marginalization for so long. Therefore, [clients] might not have the right access to information or ask the right question. I try to talk to them at their level of understanding.”*

Specialized clinics providing services to immigrants and refugees might have trained interpreters; however, their time might be limited, and they might not be available for healthcare services outside the clinics. One healthcare provider mentioned:

*“We are lucky to have interpreters in our clinic but their time is limited and most of their time is allocated for in-person appointments in the clinics and they might not be available to provide support for other program such as health promotion.”*

To achieve a positive treatment outcomes among immigrants with language barriers, effective coordination of care, good patient-provider communication and assistance with follow-up into the community post appointment are required. Lack of these ancillary services discourages individuals from accessing healthcare services. This is evident from a client’s comments:

“*I cannot speak English well and so cannot explain what I need I got so frustrated with the doctors did not go to see one in one whole year but that came to harm me. I now have pain in my ankle which is growing but what is the use of telling the doctors I cannot explain properly and they will not understand and it will not help*.”

Individuals might delay access to healthcare which increases patients’ vulnerability to adverse health outcomes.

## Discussion

This study includes the perspectives of immigrants in a Canadian city and healthcare providers serving them. Consistent with the literature, both patients and providers unanimously agreed that limited English language proficiency significantly impacts access to care, quality of care received, and health outcomes for immigrants throughout the continuum of care [3, 10, 15–17, 26–29, 31, 33]. This study examined the impacts of language barriers at all points of contact with the healthcare delivery system. The study highlights that the impacts of language barriers are evident long before an individual meets with a healthcare provider and persist long after an individual has received a treatment or intervention. The cumulative impact of this is delayed access to timely healthcare, suboptimal care, increased risk of adverse events, dissatisfaction with care received and poor health outcomes. The study emphasizes that healthcare delivery in Canada cannot be improved by providing language interpreters during medical consultation alone. A more comprehensive approach is required that includes, developing best practice guidelines for providers, training for interpreters and policy change to address the impacts of language barriers on healthcare delivery, utilization and health outcomes in Canada. This study highlights four ways in which limited English language proficiency can interfere with immigrants’ healthcare access and health outcomes.

As observed by Floyd and Sakellariou [29], clients in our study were unaware of the available healthcare services, lacked knowledge about ways to navigate the healthcare system, and were unable to advocate for needed services [25]. Language barriers impacted clients’ engagement with prevention, health promotion, and allied health services, which can create the misperception that they are disengaged in care. Other studies have also identified that language barriers influence access to and use of preventative medicine and screening [30, 42–44]. Language barriers interfere with the ability to find information about healthcare services and eligibility. This leads to fragmented, suboptimal care and/or delayed linkage with appropriate care [4, 30].

Clients and providers consistently mentioned that language barriers interfered with the development of therapeutic relationships. As observed in other studies, language barriers impeded effective health information sharing and communication between patients and providers, thereby undermining trust [16, 26–30]. Similar to what De Moissac and Bowen [38] observed, the clients in this study also mentioned difficulty describing pain and other symptoms to their healthcare providers, which can interfere with accurate diagnoses [25, 32, 45]. Clients with limited language abilities are at risk of delaying treatment [4, 38], misdiagnosis, or mismanagement of their conditions [38, 46]. Like those reported in other studies, our results also demonstrated specific instances where language barriers increased the chances of medical errors and harms due to patient’s inability to understand and/or follow treatment plans [15, 17, 25, 38].

Consistent with the findings of systematic reviews [16, 47], the providers in this study indicated that interpreters were helpful. As observed in other studies [16, 29, 30], clients in this study also emphasized the need for bilingual healthcare providers. Community health navigators can help improve access to primary and preventative healthcare services while acting as cultural brokers and language interpreters [48]. Molina and Kasper called for language-concordant care, as it has been shown to provide safe and high-quality care [49].

However, this study adds to the discussion in the literature about the challenges that arise when ad hoc interpreters are involved [50]. Consistent with the literature, the healthcare providers in this study indicated that interpreters’ roles are often unstructured. Instead of verbatim translating, an interpreter might summarize information or provide their own interpretation of what the patient and/or the provider said, leading to suboptimal conversation and care [3, 42]. Interpreters are also unsure about their role in medical translation [18]. Although healthcare providers wanted verbatim translation in our study, other studies observed that healthcare providers might expect interpreters to also act as cultural brokers or care coordinators [3, 18, 42]. Our results provided evidence of situations when some medical interpreters could not provide culturally safe translation support, especially when sensitive and taboo topics were involved [3]. Providers might not feel comfortable or prepared to care for immigrants with language barriers [25]. Language barriers may slow down conversations and additional follow-ups are required thereby increasing stress and workload for providers [27, 42, 47, 51].

In this study, clients and providers both indicated that multiple sessions might be required to communicate instructions for treatment and sample collection [42]. As observed by Ali and Watson [17] in the United Kingdom, the healthcare providers in this study also reported that interpreters might not be able to translate treatment plans, instructions for sample collection, or instructions for screenings because of their lack of medical knowledge. As discussed in the literature, the healthcare providers in this study also highlighted issues with privacy and confidentiality when ad hoc interpreters, family members, or individuals from the same ethnic groups are involved [3, 43, 50, 52]. Studies indicate that clients with limited English language proficiency prefer professional gender-concordant interpreters over family members [53]. Although studies show that without medical interpreters the quality of care is compromised for clients with language barriers, interpretation errors often occur when ad hoc interpreters are used [10, 16, 25, 26, 50, 52, 54]. Professional interpreters raise the quality of clinical care compared to ad hoc interpreters [50, 54].

Finally, the present study highlighted how English language proficiency creates an additional layer of barriers to healthcare access, utilization, and patient satisfaction [3]. Inability to communicate effectively with healthcare providers creates dissatisfaction for patients because their needs were not communicated and they are not getting the services needed [16, 27]. Moreover, language barriers limit a healthcare provider’s ability to provide care in a timely, safe manner; subsequently, the client’s needs are unmet [4, 16, 17, 27, 32].

Language barriers also create dissatisfaction for healthcare providers as they are unable to engage patients in health promotion and preventative programs [42, 44], offer additional supports like home delivery for medications, or support them with treatment adherence. Language barriers might cause embarrassment, disempower patients, and undermine patients’ confidence [25, 28, 30]. Floyd & Sakellariou [29] observed that refugee women with language barriers are likely to experience racism, and might not be engaged in healthcare decision making. Additionally, cultural belief and experience with the healthcare delivery system in the country of origin influence the type of healthcare services that will be accessed and expectation from healthcare providers [3, 28, 30]. Due to a lack of culturally appropriate care, access to healthcare services can be delayed or underutilized [12, 24, 30, 31].

Floyd and Sakellariou [29] observed that the Canadian healthcare system is organized on the assumption that service seekers can read and understand English, which marginalizes immigrants, refugees, and others with lower literacy and limited English language proficiency.

Parsons, Baker, Smith-Gorvie, and Hudak [55] mention that it is unclear who is responsible for ensuring that communication between providers and patient is adequate. Guidelines are required for healthcare providers outlining when interpreters should be involved. Papic et al. [47] highlighted the need for clear directives for determining who is responsible for arranging interpreters and finding ways to enhance the involvement of professional interpreters and multicultural clinics where available.

As a country that promotes and celebrates multiculturalism, the Canadian Charter of Rights and Freedoms (1982) guarantees equal rights, such that Canadians are to be treated with the same respect, dignity, and consideration regardless of race, nationality, ethnicity, color, religion, sex, or age [56]. Healthcare access needs to be regarded as a basic human right under the Charter and not be contingent on language proficiency. Although most immigrants arrive with better health status than the local population, largely attributed to initial health selectivity and the Canadian immigration policy, their health status tends to decline over time to levels worse than native-born citizens [3, 57–60]. This deterioration has been partly attributed to discrimination and unfair treatment that immigrants experience in the healthcare system [60].

Aery [61] proposed that a health equity perspective is required to address the socio-cultural barriers faced by vulnerable populations, including immigrants and refugees. Ali and Waston [17] proposed that addressing language barriers is an essential step towards providing culturally responsive and client-centered care. The importance of enabling patients to actively participate in their healthcare has received extensive policy attention [62]. Giving patients an active role in their healthcare empowers them and improves services and health outcomes [63]. Involving patients in shared decision making is emphasised in Saskatchewan, Canada [64]. Against this backdrop, patients, providers, and interpreters in Canada need to be engaged to understand the multi-layer barriers at the individual, community, and health-system levels and address those needs [42].

### Limitation of the study

A small number of clients from each ethnic group was recruited; therefore, results might not reflect the experience of the respective ethnic groups as a whole. With a larger number of female clients recruited in the study, the views are more reflective of female than male patients with language barriers. A small number of healthcare providers were recruited from each discipline. Further research is required to capture discipline-specific challenges encountered by providers caring for patients with language barriers. The study did not include migrant workers and refugees and additional research is required to highlight specific challenges experienced by specific groups.

### Implications for practice

The results of the study are relevant for any country accepting immigrants from linguistically diverse countries. Through professional courses, continued education, and development of best practice guidelines healthcare providers in Canada should be equipped with adequate knowledge and skills to care for patients with language barriers [49].

Interpreters in Canada should have clear instructions about whether only verbatim translation is required or they need to serve as cultural brokers and/or support clients with coordination of care. A national strategy should be developed in Canada to train, support, and supervise interpreters adequately to ensure that they deliver safe, and impactful services [35].

## Data Availability

All data generated or analysed during this study are available from the corresponding author on reasonable request.
